# Probe Contact Force Monitoring during Conductivity Measurements of the Left Atrial Appendage to Support the Design of Novel Diagnostic and Therapeutic Procedures

**DOI:** 10.3390/s22197171

**Published:** 2022-09-21

**Authors:** Hamza Benchakroun, Niko Ištuk, Eoghan Dunne, Muhammad Adnan Elahi, Tony O’Halloran, Martin O’Halloran, Declan O’Loughlin

**Affiliations:** 1 Electrical and Electronic Engineering, University of Galway, H91 TK33 Galway, Ireland; 2 Translational Medical Device Laboratory, University of Galway, H91 TK33 Galway, Ireland; 3 School of Medicine, University of Galway, H91 TK33 Galway, Ireland; 4 Aurigen Medical, Atlantic Technological University (ATU) Innovation Hub, H91 FD73 Galway, Ireland; 5 Electronic and Electrical Engineering, Trinity College Dublin, D02 PN40 Dublin, Ireland; 6 Trinity Centre for Biomedical Engineering, Trinity College Dublin, D02 PN40 Dublin, Ireland

**Keywords:** impedance sensing, conductivity, probe contact force, left atrial appendage, bio-impedance, irreversible electroporation

## Abstract

The electrical properties of many biological tissues are freely available from the INRC and the IT’IS databases. However, particularly in lower frequency ranges, few studies have investigated the optimal measurement protocol or the key confounders that need to be controlled, monitored, and reported. However, preliminary work suggests that the contact force of the measurement probe on the tissue sample can affect the measurements. The aim of this paper is to investigate the conductivity change due to the probe contact force in detail. Twenty ex vivo bovine heart samples are used, and conductivity measurements are taken in the Left Atrial Appendage, a common target for medical device developments. The conductivity measurements reported in this work (between 0.14 S/m and 0.24 S/m) align with the literature. The average conductivity is observed to change by −21% as the contact force increases from 2 N to 10 N. In contrast, in conditions where the fluid concentration in the measurement area is expected to be lower, very small changes are observed (less than 2.5%). These results suggest that the LAA conductivity is affected by the contact force due to the fluid concentration in the tissue. This work suggests that contact force should be controlled for in all future experiments.

## 1. Introduction

The electrical properties of biological tissues have been of interest since 1894 and have been widely studied in the literature [1,2]. The electrical properties of biological tissue have been studied for several human organs [3,4,5,6,7,8,9,10,11] and have been used to develop many therapeutic and diagnostic technologies [12,13,14,15,16,17,18,19]. 

The measured electrical properties have been synthesized into several freely available databases, which can be accessed online, including the Italian National Research Council database [20] and the IT’IS Foundation database [21]. Despite the substantial details and information about the sources in the online databases, in many cases, differences exist between the acquisition hardware, protocols, and associated metadata of the original data. The databases present continuous data between 10 Hz and 100 GHz, even though, in many cases, these data are acquired using different techniques in different studies, even for the same tissue type [21]. 

Many data acquisition approaches are presented in the literature, but few studies have examined the optimal measurement protocol or determined the important confounders that should be controlled, measured, and recorded. These issues are particularly prevalent below 100 kHz, as fewer studies are available in this frequency range. Hence, the synthesized data can frequently change as new studies become available. In recent versions of the online databases, the reported conductivity has changed by a factor of two for several tissues. A large and recent change in reported conductivity has occurred for bone (cortical), brain (grey matter), brain (white matter), eye (vitreous humor), fat, medulla oblongata, midbrain, muscle, pons, spinal cord, and thalamus tissues [21]. These changes in the properties reported in the literature in recent years alone suggest that further work is required to investigate the electrical properties below 100 kHz [21,22,23,24,25]. 

For example, it was noted by the IT’IS Foundation during an update of the Tissue Properties Database in February 2022 [21,24,25] that several tissues have changed substantially. These changes in reported electrical properties below 100 kHz could have impacted previous therapeutic and diagnostic technologies. Thus, research regarding methods to accurately acquire and report electrical properties below 100 kHz remains of interest, and improvements may be of use to medical device designers.

The electrical properties (in terms of conductivity, *σ*) are acquired using a probe in contact with the tissue to measure the ratio of electrical charge flow in response to a difference in electrical potential. The measuring methods are reviewed in detail in [6,23,26,27,28]. The contact between the probe and tissue is fixed throughout the experiment. However, there is no clear understanding regarding the effect of the contact force of the probe on the tissue on the resulting measurements [5,29,30]. Furthermore, the mechanisms behind the change in conductivity due to contact force are not fully understood, and the effect of contact force on tissue conductivity varies between tissue types [5,30,31,32]. 

However, it is known that the conductivity can vary with the contact force. This confounder cannot be neglected, particularly in diagnostic and therapeutic procedures for complex sensitive tissue. For this study, the left atrial appendage (LAA) of the heart is used as a test case to evaluate the effect of contract force on conductivity. Typically, studies consider the heart as one region without reporting the properties of individual areas or the area of measurement, so data on the LAA conductivity is very limited. However, the LAA is a common target for medical device development as the LAA has been identified as a frequent source of aberrant impulses in the heart that cause Atrial fibrillation (AF) [33,34,35]. 

The limited availability of data and the known issues regarding the impact of the contact force on the measurements motivated the study in this paper. The main aim of this paper is to investigate the frequency-dependent conductivity of the LAA with respect to the contact force of the probe in the range of 2 N to 10 N in the frequency range from 10 Hz to 100 kHz. Potential causes of conductivity variance and the acquired data are statistically compared to investigate if the extracellular fluids are contributors to the conductivity change.

The results of this paper will help improve the understanding of the tissue conductivity measured under different contact forces and could be broadly applicable to the design and optimization of novel diagnostic and therapeutic procedures. Some of therapeutic and diagnostic technologies that can be impacted are electromagnetic dosimetry studies [12,13], pulsed-field ablation (electroporation) [14], electrical impedance spectroscopy [15,16], electrical impedance tomography (EIT) [17,18], tissue engineering and treatment monitoring [19].

The remainder of this paper is structured as follows. Section 2 reviews the current literature on conductivity measurement and the influence of the contact force on conductivity. Section 3 describes the methodology of this study, including the electrical properties acquisition process, setup design and sample handling. Section 4 presents the results, assesses the influence of contact force on the conductivity acquired from twenty ex vivo LAAs and statistically analyses the significance of extracellular fluids as a contributing factor to the effects observed. The conclusion and future work are reported in the closing Section 5.

## 2. Background

Recently, non-thermal pulsed-field ablation using electroporation has been proposed as a treatment for AF [36], and the electrical conductivity of the LAA is used in treatment planning [37,38,39]. Non-thermal pulsed-field ablation causes changes in the cell wall of the targeted tissue using bursts (order of ns to µs) of high voltage pulses (order of kV). Depending on the applied pulses, pulsed-field ablation can cause permanent ablation of the tissue, which can be used to remove aberrant conduction pathways in the heart to help treat AF [40,41]. Therefore, the treatment planning for non-thermal pulsed-field ablation often exploits knowledge of LAA conductivity at lower frequencies.

The conductivity of biological tissues at lower frequencies (specifically below 100 kHz) is poorly studied but important. For example, these conductivity values are required for non-thermal pulsed-field ablation device design and treatment planning [42]. Measuring conductivity below 100 kHz is challenging, and achieving high accuracy and repeatability is difficult due to the nature of the stimulus [2,25,42]. Electrical currents below 100 kHz typically flow in the extracellular spaces: the charges do not penetrate the cell membrane but flow around the cells. By contrast, higher frequency currents (typically above 100 kHz) flow freely through living tissues (extracellular flow), making sensing at the cellular level infeasible [42]. Therefore, the conductivity below 100 kHz is affected by the cellular level of the tissue.

Furthermore, several confounding factors can impair the measurement accuracy, such as the sample type, sample handling, the sample size [43], hydration levels [30], and the source of the sample [44]. The contact force between the probe and sample [45], inaccuracies in calibration [46], the heterogeneity of the sample [28], and inconsistencies in temperatures [46] are additional confounders introduced during the measurement process. These sources of error plus the importance of analyzing the influence of each on the data acquired are reviewed in detail in [47].

Studies of biological tissues typically use the 4-electrode method to acquire the conductivity, using two electrodes to inject the alternating current and two electrodes to measure the electrical potential. The ratio between the injected current and the measured electrical potential is the complex impedance. The conductivity is then calculated from the complex impedance.

Using two separate pairs of electrodes for the current injection and the voltage measurement in the 4-electrode method is preferred as it shows improved performance compared with 2- or 3-electrode methods, which share electrodes [48,49,50]. The 4-electrode method displays low electrode polarization influence and lower charging currents at the electrode interface [48,49,50].

The tetrapolar probes used for the 4-electrode method can be designed in many ways depending primarily upon the geometry and spacing of the electrodes, the type of the electrodes (planar electrodes, needle electrodes, pin electrodes), and the electrode material [9,10,51,52,53]. In the literature, the 4-electrode method is commonly used, and conductivity values in the frequency range below 100 kHz are commonly acquired using the 4-electrode method. Nowadays, researchers often rely on online databases to get the values of the electrical properties of more than 112 tissues from 10 Hz to 100 GHz [7,22], which are widely cited (more than 1700 citations). However, in the source data, the probe contact force is typically not controlled, not monitored, and not reported. If the contact force is reported, the contact force will be qualitatively described as “sufficient contact” or on a scale from “light contact” to “moderate” to “firm” [5,29,30]. Of the ten studies reporting the conductivity of heart muscle in the IT’IS Foundation database, only three studies describe the contact force used [21]. Table 1 shows these ten heart muscle studies and how the contact pressure during the measurement is described.

In addition to quantitative reporting of the contact force, the effect of the contact force on the tissue conductivity may vary depending on the tissue type. Of the studies which have investigated the effect of contact force on the measured conductivity of tissue, different trends have been reported, from increasing conductivity with increased force to decreasing conductivity with increased force [5,30,31,32]. These studies investigating tissue conductivity change due to contact force do not appear in the online databases at the time of submission. 

The applied contact force has a direct effect on the properties of biological tissue [58]. The applied force can lead to internal extracellular fluids movement, deformation of the tissue, or tissue damage [58]. The tissue extracellular fluids movement in the biological tissue is commonly referred to as the main contributor to the tissue conductivity change, assuming that the biological tissue is measured without any mechanical damage [5,59]. Monotonically increasing the contact force on the tissue decreases the measured conductivity due to the movement of extracellular fluids away from the measuring area and that can be seen in the cervix as increasing the contact force on the probe displaces a high conductance mucus film on the cervix thus effectively decreasing tissue electrical conductivity [5,59]. However, for some tissues, such as lung, the conductivity is increased as more contact force is applied, which is likely due to the air being pushed away from the alveoli [32]. Furthermore, different behavior is seen in liver tissue, where the contact force applied by the probe has little effect on the conductivity [31]. A change of +0.8% per 1 N of applied force (per 6.25 kPa of applied pressure) is observed in the liver [31]. Table 2 shows the different behavior of the various tissues under monotonically increasing contact force.

The different trends of tissue conductivity change with respect to contact force in different tissues helps to motivate this work. This paper investigates the LAA conductivity change with respect to varying contact forces between 2 N and 10 N between 10 Hz and 100 kHz. Furthermore, different protocols are investigated to help determine the causes of the changes in the measured conductivity.

## 3. Materials and Methods

In this study, we examine the effect of the contact force applied by the tetrapolar probe on the measured LAA conductivity and assess if the extracellular fluids concentration in the tissue is a contributing factor. Three related experiments are performed: the forward experiment, the reverse experiment, and the hyperhydration experiment. 

### 3.1. Forward Experiment

The forward experiment measures the LAA conductivity at different contact forces between 2 N and 10 N at +1 N intervals, where the contact force is increased monotonically between each measurement. The forward experiment is conducted on ten ex vivo LAA bovine samples. The initial force 2 N was chosen to have sufficient contact between the LAA and the probe [5]. A force of above 10 N is typically higher than would be experienced by the tissue in vivo [39,59]. For each sample, the conductivity is measured at the pectinate muscle in the opening of the LAA.

### 3.2. Reverse Experiment 

The reverse experiment measures the LAA conductivity at different contact forces between 10 N and 2 N at −1 N intervals where the contact force is first increased to 10 N, and then measurements are repeated as the contact force is reduced back to the starting value of the forward experiment (2 N). By reversing the order of the measurements, this experiment examines if the changes observed in the forward experiment are due to some change in the tissue conductivity due to the contact force. Five ex vivo LAA samples were used, and the same procedure as the forward experiment was used.

It has been suggested in the literature that the contact force can cause the concentration of the extracellular fluid in the tissue to change, which would lead to differences in the forward and reverse experiments. However, as the contact force is decreased, there may be a partial increase in fluids in the measurement area due to tissue relaxation [60]. Thus, the hyperhydration experiment was designed to cover the limitation of partial extracellular fluids flowing back into the measuring area.

### 3.3. Hyperhydration Experiment

The hyperhydration experiment is conducted to monitor the LAA conductivity change with respect to contact force after the sample has been preprocessed to reduce the extracellular fluid concentration. As the extracellular fluid is ionic [61], reducing the extracellular fluid content of the LAA reduces the number of charge carriers, which would decrease the change of measured conductivity due to extracellular fluids movement in response to the measurement contact force. Constant conductivity measurements with respect to contact force in this experiment would suggest that extracellular fluids movement is a contributor to the effects observed in the forward experiment.

For the hyperhydration experiment, each of the five samples is first submerged in deionized (DI) water for one hour. This process is to reduce the ion content of the extracellular fluid [62]. After this process, the sample is removed from the water, and the experiment proceeds as per the forward experiment.

### 3.4. Data Acquisition 

**Figure 1:**
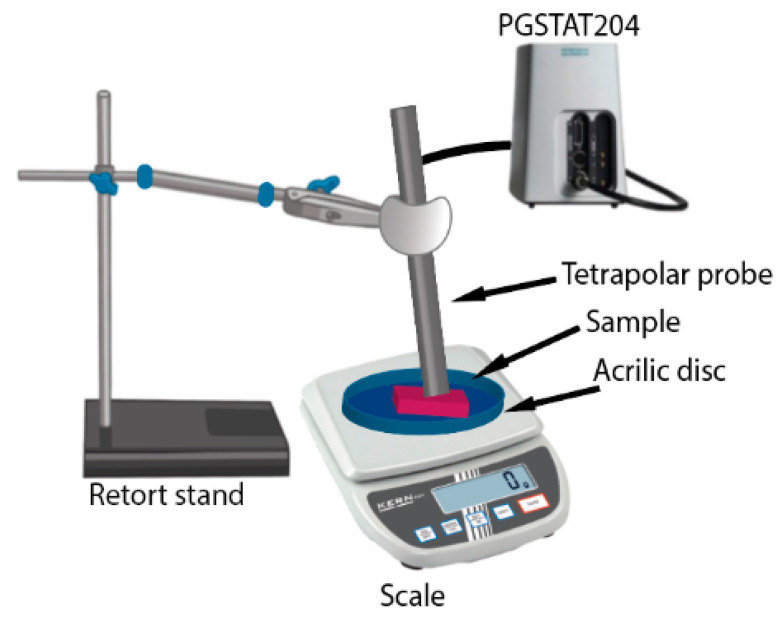
Measurement setup used to monitor the conductivity with the probe, the PGSTAT204 Galvanostat and retort stand used to lift and keep the probe fixed and the weighing scale.

The conductivity is acquired using a PGSTAT204 (Autolab, Kanaalweg Den-Haag, The Netherlands) in Galvanostat mode (100 µA) at room temperature with Nova 2.1 software. The conductivity is measured for the targeted frequencies 10 Hz, 100 Hz, 1 kHz, 10 kHz, and 100 kHz. The measurement setup consists of the PGSTAT204 connected to the tetrapolar probe (i.e., working electrode, counter electrode, reference electrode, and sensing electrode), the PGSTAT204 connected to a computer that uses the Nova 2.1 to control the PGSTAT204, a weighing scale (Kern Weighing Scale, METTLER TOLEDO, Columbus, OH, USA) to monitor the contact force applied on the LAA. The setup is shown in Figure 1.

The conductivity is acquired from the frequency-dependent complex impedance using the methods described in [6,26,27]. The conductivity is then computed from the measured frequency-dependent complex impedance and the probe cell constant using Equation (1) [6,26].

(1) σ=G×k

where *σ* [Sm^−1^] is the electrical conductivity of a reference liquid, *G* [S] is the measured conductance (derived from the complex impedance *Z*), and *k* [m^−1^] is the cell constant. *k* = 0.032 m^−1^ for the proposed tetrapolar probe in this study for the frequency ranges from 10 Hz to 100 kHz. The Pearson correlation coefficient for *k* is *R*^2^ = 0.99 between the measured conductance and the reference conductivity. 

The tetrapolar probe in this study was designed to maximize the potential accuracy for measurements in the LAA. Firstly, a common typology of a collinear tetrapolar probe with equal spacing between electrodes is used. The voltage is measured between the inner electrodes, and the current is injected using the outer electrodes. This collinear configuration is commonly used in the literature [9,10,51,52,53]. The inner measuring electrodes are in the region of maximum current density, resulting in high probe sensitivity [9,10,51,52,53]. Due to the limitations of the measuring area in the LAA, a small probe footprint was desired, which is mainly determined by the electrode size and the electrode spacing.

**Figure 2:**
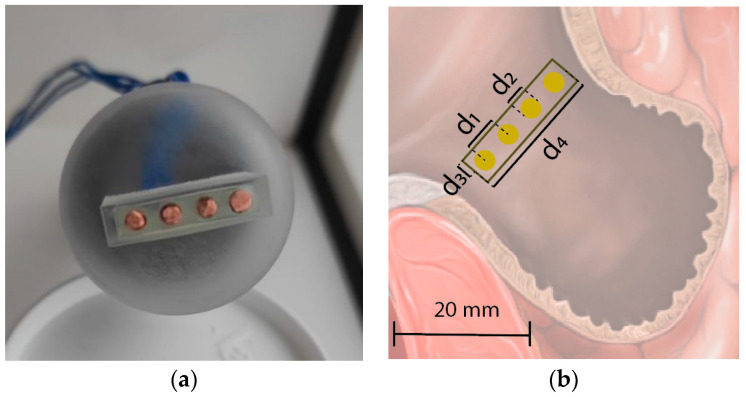
(**a**) Photo of the manufactured prototype of the proposed tetrapolar collinear probe; (**b**) Diagram showing the dimensions of the used tetrapolar collinear probe. The illustration shows the footprint of the probe on the surface of the LAA.

Small electrodes are desired, yet electrodes smaller than 1 mm in diameter can introduce undesired effects such as non-linearities, increased electrode impedance, and increased electrode polarization. Similarly, small electrode spacing is desired, but the tetrapolar probe sensing depth is proportional to the distance between the inner electrodes. In this work, the smallest suitable electrodes were chosen (1 mm diameter), and the electrode spacing was chosen to match the depth of the LAA of 5 mm. The total footprint of the tetrapolar probe used in this study is 20 mm by 4 mm. The prototype of the tetrapolar probe is shown in Figure 2a, and a schematic representation overlaid on the LAA in Figure 2b, where the probe dimensions are *d*_1_ = 5 mm, *d*_2_ = 3 mm, *d*_3_ = 2 mm, *d*_4_ = 20 mm.

### 3.5. Force Monitoring 

The applied contact force by the probe is monitored using a weighing scale placed under the sample; the required probe contact force is derived from the mass recorded by the scale as follows:

(2) F=m×g

where *F* [N] is the weight force applied by the probe, *m* [kg] is the mass recorded by the weighing scale (0.1 g accuracy) after been tared and *g* is the acceleration due to gravity. The acceleration due to gravity in Galway, Ireland, where the measurements are performed, is equal to 9.801 m/s^2^. For example, to ensure a 2 N applied contact force on the LAA by the probe, a mass of 0.20406 kg should be measured. Due to the semi-solid nature of the samples and the finite measurement time, the sample can deform during measurement, which may result in a change in the contact force. Hence it is important to monitor the contact force during and after measurement.

The measurement protocol for monitoring the contact force was as follows:

- Firstly, the sample was placed on the weighing scales, and the scale was tared.
- The probe was placed in contact with the sample and adjusted until the mass read the appropriate value for the desired probe contact force as per (2).
- The mass was monitored during the conductivity measurement, and if the mass varied by more than 2% over the course of the conductivity measurement, the measurement was repeated.

### 3.6. Statistical Analyses

To investigate the significance of the conductivity changes in this study between the three different experiments, two T-tests are conducted. The T-tests compare the conductivity change between the forward experiment with a high extracellular fluid concentration in the measurement area and the conductivity change in the experiments with a lower extracellular fluid concentration in the measurement area (the reverse and hyperhydration experiments). The first T-test (T1) tests against the alternative hypothesis that the conductivity change due to the contact force of the forward experiment is larger than the conductivity change due to the contact force of the reverse experiment. If T1 shows a significant difference, this is consistent with the theory that fluid concentrations are a contributor to the observed differences.

The second T-test (T2) tests against the alternative hypothesis that the conductivity change due to the contact force of the forward experiment is larger than the conductivity change due to the contact force of the hyperhydration experiment. Similarly, if T2 shows a significant difference, this is also consistent with the theory that fluid concentrations are a contributor to the observed differences.

Both T1 and T2 are conducted at a 5% significance level, and a *p*-value less than 1% is considered significant (*p* < 0.01) [63,64,65]. The tests were conducted for each frequency individually, as the variation of conductivity is frequency-dependent.

### 3.7. Tissue Handling 

Measurements were performed on twenty ex vivo bovine LAA samples in total: ten samples for the forward experiment, five for the reverse experiment, and five for the hyperhydration experiment. The bovine hearts were obtained from a local slaughterhouse immediately after excision and transported to our laboratories in vacuum-sealed containers. All samples arrived at the laboratories within two hours of excision. The twenty hearts were embedded in their fat capsule to limit the dehydration of the tissue. The LAA was dissected from the heart, and the internal membrane was removed to measure directly on the LAA pectinate muscle. Measurements were performed on the endocardium of the LAA, where the electroporation treatment usually occurs. 

**Figure 3:**
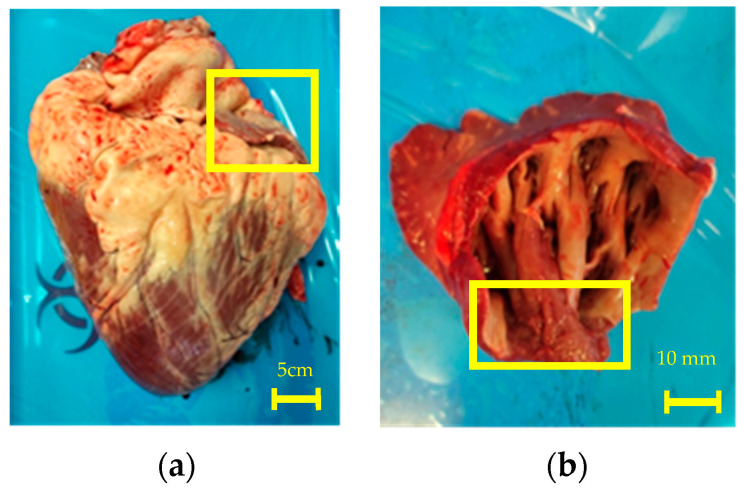
(**a**) Left picture shows the first sample bovine heart with the LAA highlighted; (**b**) The right picture shows the dissected LAA from sample one. The yellow square shows the position of the LAA pectinate muscle where the measurements were taken.

The dissected LAA and its position in the heart can be seen in Figure 3a. All experiments were conducted at the opening of the LAA at the position shown in Figure 3b. The temperature was monitored during all experiments. The initial and final temperature was within the range of 20 ± 0.5 °C for all samples.

To validate the measurement setup, three pre- and post-validation measurements with 0.15 M NaCl solution were acquired to measure any drift in the measurement setup.

## 4. Results

To our knowledge, the conductivity of the LAA specifically is not available in the literature. Therefore, the measured LAA conductivity in this paper is compared with cardiac muscle values from the literature: 0.05 S/m to 0.9 S/m. The conductivity range from the literature is large, as these values are from studies with different tissues (different animals or human), both in vivo and ex vivo, and at different locations in the heart [7,9,15,51,54,55,56,66,67].

**Figure 4:**
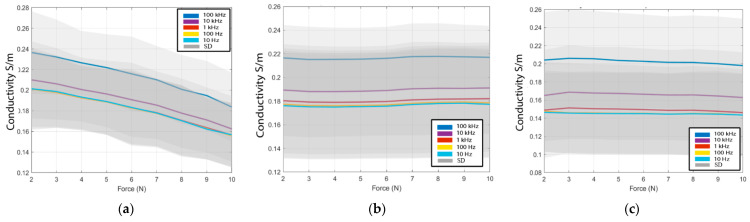
The average variation of the conductivity with the contact force for (**a**) forward experiment (**b**) reverse experiment (**c**) hyperhydration experiment plus the standard deviation (grey cloud) trend at 10 Hz, 100 Hz, 1 kHz, 10 kHz, and 100 kHz.

Figure 4 shows the average conductivity and standard deviation from the forward, reverse, and hyperhydration experiments at 10 Hz, 100 Hz, 1 kHz, 10 kHz, and 100 kHz with respect to contact force. The average conductivity values at all contact forces are in the range of the literature values, varying from a minimum of 0.15 S/m in the hyperhydration experiment to a maximum of 0.24 S/m at low contact force in the forward experiment. The standard deviation is high for all three experiments at ±14%. As reported, the drift error is less than +2% for all measurements, suggesting that the high standard deviation is due, for the most part, to the differences between the samples and the probe-sample contact and not due to errors in the acquisition hardware sensitivity of the tissue in frequency below 100 kHz [25,42]. This variance is primarily due to the change between samples is mostly introduced by tissue properties, as the absolute magnitude of drift error is less than +2% from the pre- and post-validation measurement for all experiments.

**Figure 5:**
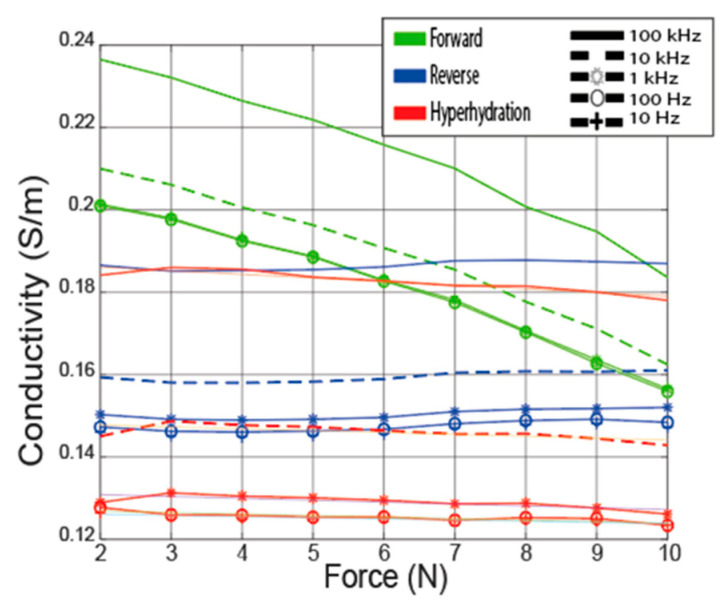
The average variation of the conductivity with the force for the forward, reverse, and hyperhydration experiment at 10 Hz, 100 Hz, 1 kHz, 10 kHz, and 100 kHz.

The average conductivity acquired from the 10 samples from the forward experiment, 5 samples from the reverse experiment, and the 5 samples from the hyperhydration experiment are computed and then compared. The Pearson coefficient (*R*^2^) of the average conductivity with the total data set is higher than 0.99 for the forward, reverse, and hyperhydration experiments. Pearson coefficient higher than 0.99 shows that the average conductivity is a good representation of each sample set. Figure 5. shows the summary of all the average conductive from the forward, reverse, and hyperhydration experiments for the targeted frequencies 10 Hz, 100 Hz, 1 kHz, 10 kHz, and 100 kHz. 

Comparing the slopes of fitted lines shown in Table 3 of the LAA conductivity in the reverse experiment and the hyperhydration experiment with the forward experiment, it is observed that the change in the forward experiment is much higher than the change in two other experiments. We statistically analyze the significance of the change using the method displayed in the statistical analyses section. The *p*-values from the T1 and T2 are displayed in Table 4 and are <0.01, reflecting that the change seen in the forward experiment is significantly higher than the change seen in the reverse and hyperhydration experiments.

The conductivity for the forward experiment is observed to decrease due to monotonically increasing contact force with a slope of −6.5 × 10^−3^ S/(m × N). The average decrease of the conductivity due to the increase of the contact force during the forward experiment (−21%) is in the same range of change seen in the cervix ([5] in Table 3). The LAA and cervix are both identified as muscle tissue, and similar behavior is expected; increasing the contact force of the probe on the tissue decreases the fluid concentration in the measurement area.

The conductivity changes due to contact force seen in the reverse experiment are observed to be small. The slope of the conductivity variation of the reverse experiment is equal to 2.64 × 10^−4^ S/(m × N). Similarly, the slope of conductivity variation of the hyperhydration experiment is small and equal to −8.8 × 10^−4^ S/(m × N). These results support the theory that the changes due to the contact force are due to changes in fluid concentration in the measurement area.

**Figure 6:**
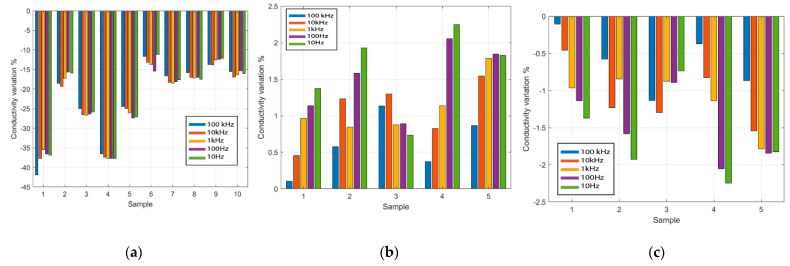
The percentage variation of the conductivity from the proposed experiment at 10 Hz, 100 Hz, 1 kHz, 10 kHz, and 100 kHz. (**a**) Forward experiment with ten LAAs, contact force from 2 N to 10 N (**b**) Reverse experiment with five LAAs, contact force from 10 N to 2 N (**c**) the hyperhydration experiment with five clarified LAAs, contact force from 2 N to 10 N.

Figure 6a shows the percentage variation of the conductivities of each sample in the forward experiment from the initial force of 2 N to the final force of 10 N at frequencies 10 Hz, 100 Hz, 1 kHz, 10 kHz, and 100 kHz. The average variation of the conductivity between the ten samples is equal to −21% between the initial and the initially applied force of 2 N, and the final applied force of 10 N. Figure 6b shows the results of the reverse experiment from the initial force 10 N to the final force 2 N at frequencies 10 Hz, 100 Hz, 1 kHz, 10 kHz, and 100 kHz. The conductivity is observed to have an average change of +1.3% due to the contact force from the five LAA measured. Figure 6c shows the percentage variation of the conductivities of the hyperhydrated LAA samples (with extracellular fluids partially removed) from the initial force from 2 N and the final force of 10 N at the frequencies 10 Hz, 100 Hz, 1 kHz, 10 kHz, and 100 kHz. The average conductivity is observed to change by −2.5% in the five clarified LAA samples.

Interestingly, the conductivity change in each set of samples is different. For example, in the forward experiment, the maximum and the minimum conductivity changes due to contact force are seen in sample 1 and sample 6, with a −41% decrease for sample 1 and −12% for sample 6. The change in conductivity due to contact force could be related to the initial sample extracellular fluid concentration, which is not always known in ex vivo samples. The higher the initial fluid concentration, the larger a change would be expected.

In summary, the conductivity decreases as the contact force increases. These changes are significant when compared with the smaller changes observed in the reverse and hyperhydration experiments. 

## 5. Conclusions

In this work, we present conductivity measurements of twenty ex vivo bovine hearts, specifically the LAA, between 10 Hz and 100 kHz. The contact force of the measurement probe on the sample is controlled, monitored, and reported, and the measurements are repeated for nine different forces between 2 N and 10 N. The measured conductivity values are between 0.14 S/m and 0.24 S/m, which align with the values reported in the literature for cardiac muscle. Additionally, an average change of −21% in conductivity was observed as the contact force increased from 2 N to 10 N.

Secondly, the potential causes of the observed change in conductivity were examined. Previous work has suggested that extracellular fluids concentration in the measurement area is a key factor in the changes in measured conductivity due to changing contact force. The twenty samples were split into three experiments: the forward experiment, reverse, and hyperhydration experiments. In the forward experiment, contact force was increased monotonically. In the reverse experiment, the contact force was first increased to the maximum and the conductivity measured as the contact force was decreased. The hyperhydration experiment was the same as the forward experiment, except the tissue was first preprocessed to reduce the extracellular fluid content of the whole sample.

Compared with the average change of −21% in measured conductivity in the forward experiment, an average change of +1.3% and −2.5% of conductivity was observed in the reverse and hyperhydration experiments. These results suggest that the initial high contact force in the reverse experiment decreased the extracellular fluids content in the measurement area, leading to minimal change in the conductivity across the contact force range. Similarly, the much smaller change observed in the hyperhydration experiment suggests that the ionic extracellular fluids removed during the processing of the samples were partially responsible for the change in conductivity due to the contact force observed in the forward experiment.

Moreover, these results suggest that the contact force of the probe in tissue conductivity measurements should be controlled, monitored, and reported. A number of methods exist for controlling the contact force, but the setup used in this work is both easy-to-use and accurate. More complex methods exist, such as using a strain gauge or other sensor to control the contact force. 

Finally, these results suggest that more research is needed into the electrical properties of biological tissues in lower frequency ranges below 100 kHz. Specifically, future studies should look at standardizing the measurement protocol and reporting standards to increase the quality and repeatability of the conductivity measurements in the literature.

## Figures and Tables

**Table 1:** Contact force reported by the ten studies investigating heart muscle conductivity from the IT’IS Foundation database and how the contact pressure during the measurement is described.

	Year	Species	Number	Ex Vivo	In Vivo	Force
[54]	1967	Various	-	✓	✓	-
[55]	1980	Dog	7	✓	✓	-
[51]	1980	Dog	4		✓	Gentle pressure
[53]	1987	Dog	12		✓	-
[56]	1993	Sheep	39		✓	-
[57]	1995	Pig	10		✓	Electrodes were 4 to 6 mm deep
[22]	1996	Various	>30	✓		Firm
[52]	1997	Pig	26		✓	-
[23]	2002	Pig	8		✓	-
[9]	2009	Pig	>3		✓	-

**Table 2:** Percentage change of the electrical properties of cervix, lung, and liver with monotonically increasing contact force.

Tissue	Force Range	% Change in Electrical Properties
Cervix [5]	Soft to firm	−21.7%
Lung [32]	1 N to 10 N	+44.9%
Liver [31]	2.9 N to 29 N	−7%
Liver [45]	1 N to 10 N	−8%

**Table 3:** The measured conductivity changes by −21% and −2.5% due to the increase of force contact for the set of samples in the forward and the hyperhydration experiment, respectively, +1.3% change is seen in the conductivity in the samples in the reverse experiment.

Experiment	y-Intercept [S/m]	x-Intercept [N]	Slope [S/(m × N)]	*σ* Change (%)
Forward	0.239	0.187	−6.45 × 10^−3^	−21%
Reverse	0.185	0.187	2.64 × 10^−4^	+1.3%
Hyperhydration	0.186	0.179	−8.8 × 10^−4^	−2.5%

**Table 4:** *p*-Values from the two tests conducted to assess the significance of the variation between the forward experiment, the reverse, and the hyperhydration experiment.

	Frequencies				
	10 Hz	100 Hz	1 kHz	10 kHz	100 kHz
*p*-value T1	0.23 × 10^−3^	0.08 × 10^−3^	0.088 × 10^−3^	0.13 × 10^−3^	0.25 × 10^−3^
*p*-value T2	0.59 × 10^−3^	0.19 × 10^−3^	0.24 × 10^−3^	0.34 × 10^−3^	0.48 × 10^−3^

## Data Availability

Not applicable.
